# Interactive effects of nitrogen rates and deficit irrigation on yield, forage quality, and water productivity of silage sorghum under subsurface drip irrigation

**DOI:** 10.3389/fpls.2026.1832758

**Published:** 2026-07-29

**Authors:** Mehmet Alagöz, Mevlüt Türk

**Affiliations:** Faculty of Agriculture, Department of Field Crops, Isparta University of Applied Sciences, Isparta, Türkiye

**Keywords:** crop water consumption, deficit irrigation, nitrogen rates, subsurface drip irrigation, water use efficiency

## Abstract

This study was conducted to determine the effects of irrigation levels and nitrogen applications on the yield and quality of sorghum (*Sorghum bicolor* L.) during the years 2021 and 2022 in Isparta, located in the Mediterranean region of Türkiye. The Greengo variety was used in the study. Under a subsurface drip irrigation system, six irrigation treatments representing 0 (rainfed), 25, 50, 75, 100, and 125% of reference evapotranspiration (ET_0_) replenishment and five nitrogen rates (0, 80, 160, 240, and 320 kg N ha^-1^) were applied. The experiment was conducted as a split-plot arrangement in a randomized complete block design with three replications. Measurements included Ky (yield response factor), crop water consumption, WUE (water use efficiency), IWUE (irrigation water use efficiency), plant height, number of leaves, stalk thickness, leaf/stalk ratio, green forage yield, dry matter yield, crude protein content (CP), crude protein yield, NDF (neutral detergent fiber), ADF (acid detergent fiber), crude ash, phosphorus, potassium, calcium, magnesium, leaf greenness (SPAD), and stomatal conductance. Irrigation levels and nitrogen rates significantly affected all measured traits. The Ky ranged from 0.805 to 1.479, crop water consumption from 394.2 to 795.6 mm, plant height from 185.0 to 327.7 cm, green forage yield from 31.91 to 114.76 t ha^-1^, crude protein content from 5.94% to 7.23%, NDF from 46.37% to 57.77%, and ADF from 25.83% to 35.93%. Overall, full irrigation (I100) combined with 240 kg N ha⁻¹ provided the best overall balance between forage yield, forage quality, and irrigation water use efficiency and is therefore recommended as the optimum management strategy for silage sorghum production.

## Introduction

Increasing water scarcity has intensified the search for forage crops capable of maintaining high productivity under deficit irrigation. Sorghum (*Sorghum bicolor* L.) is considered one of the most drought-tolerant forage crops because of its high adaptability to adverse environmental conditions (Rooney, 2004). Compared with conventional forage crops such as maize, sorghum requires less water and maintains relatively high productivity under limited water conditions, making it a promising alternative for sustainable forage production in arid and semi-arid regions (Farre and Faci, 2006). In recent years, increasing drought frequency, irregular precipitation patterns, and climate change-related water scarcity have further increased the importance of sorghum as a climate-resilient forage crop (Osakabe et al., 2014; Abd El-Mageed et al., 2018).

Agricultural production accounts for a substantial proportion of global fresh water consumption, and improving water use efficiency has become one of the major priorities for sustainable crop production. Deficit irrigation strategies have therefore gained increasing attention, particularly in water limited regions. Reported that water deficit significantly affects sorghum growth, biomass production, forage quality, and physiological responses (Aydinşakir et al., 2018; Kaplan et al., 2019; Allamine et al., 2023). Among modern irrigation techniques, subsurface drip irrigation has emerged as an efficient method that minimizes evaporation and surface runoff losses by delivering water directly to the root zone (Ayars et al., 1999; Camp et al., 2000). Subsurface drip irrigation may also enhance nutrient uptake efficiency and reduce environmental losses associated with fertilization. Recent studies have demonstrated that optimized irrigation management can improve water productivity and maintain acceptable forage yield and quality in sorghum under drought conditions (Kaplan et al., 2019; Varol et al., 2025).

Nitrogen is one of the most important nutrients limiting crop productivity and forage quality in sorghum production systems. Nitrogen also plays a critical role in chlorophyll synthesis, photosynthetic activity, protein accumulation, and plant growth, directly affecting biomass production and forage nutritive value (Schaper and Chocko, 1997; Ullah et al., 2015). Adequate nitrogen supply has been reported to increase plant height, leaves area, chlorophyll content, crude protein concentration, and overall forage productivity in sorghum (Ajeigbe et al., 2018; Sadighfard and Geren, 2022). In contrast, excessive nitrogen applications may reduce nitrogen use efficiency and increase environmental risks through nitrate leaching and nutrient losses (Polat, 2022). Moreover, nitrogen availability strongly influences physiological processes associated with drought tolerance, including stomatal conductance, photosynthetic efficiency, and osmotic regulation under water deficit conditions (Kabay and Şensoy, 2016).

Forage quality is another important criterion when evaluating sorghum for animal feeding. Parameters such as crude protein (CP), neutral detergent fiber (NDF), acid detergent fiber (ADF), and mineral composition directly influence forage digestibility, feed intake, and animal performance (Yang and Beauchemin, 2009). Previous studies have shown that irrigation and nitrogen management can substantially alter forage quality traits in sorghum by affecting structural carbohydrate accumulation and nutrient composition (Kilcer et al., 2002; Kaplan et al., 2019). Water deficit conditions may increase crude protein concentration while reducing fiber fractions, whereas excessive vegetative growth under high irrigation conditions may increase lignification and reduce digestibility (Newman, 2014). Furthermore, irrigation and nitrogen availability may significantly affect mineral uptake and physiological traits such as chlorophyll content and stomatal conductance, which are important indicators of plant adaptation to environmental stress conditions (De Souza et al., 2015; Allamine et al., 2023).

Although numerous studies have investigated the separate effects of irrigation or nitrogen fertilization on sorghum production, studies evaluating the interactive effects of deficit irrigation and nitrogen management under subsurface drip irrigation systems remain limited. In particular, integrated evaluations combining yield, forage quality, mineral nutrition, water use efficiency, and physiological responses under different irrigation and nitrogen combinations are still insufficient. Therefore, this study aimed to determine the effects of different irrigation levels and nitrogen rates on yield, forage quality, mineral composition, water use efficiency, and physiological characteristics of sorghum grown under subsurface drip irrigation conditions and to identify optimal irrigation and nitrogen management strategies for sustainable forage production.

## Materials and methods

### Climate and soil characteristics of the experimental site

**Figure 1:**
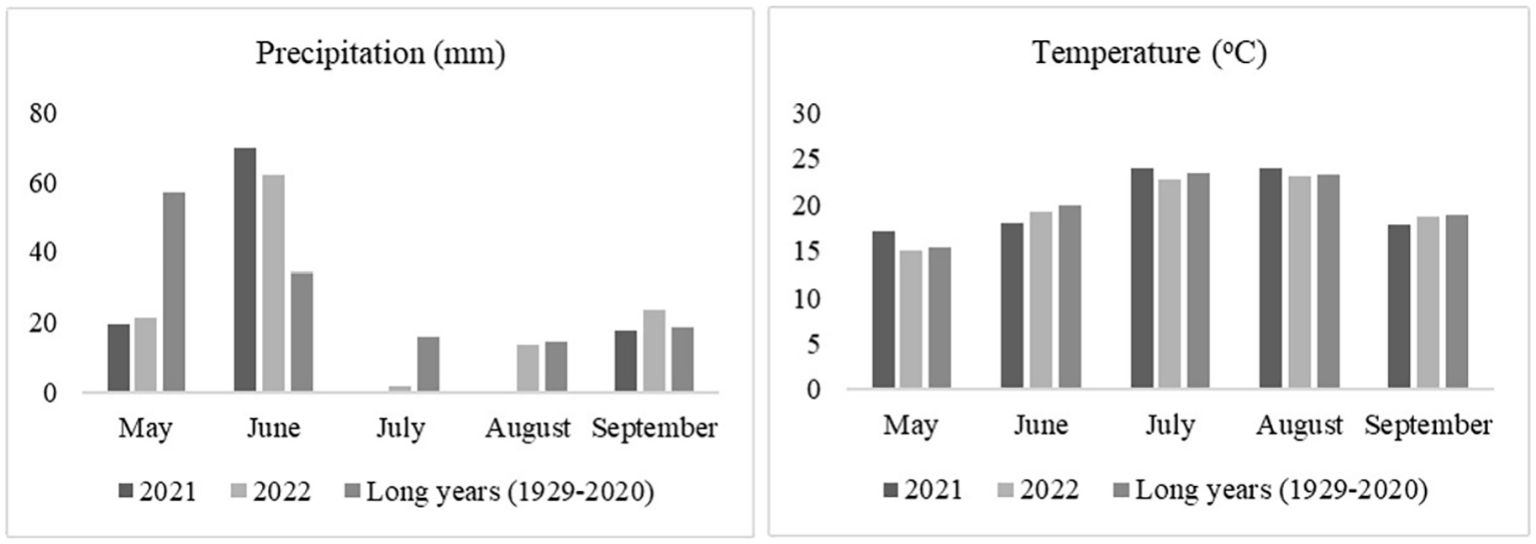
Monthly rainfall and temperature data for the experimental area.

The research was conducted in Isparta Province (37°45’ N, 30°33’ E; elevation 1035 m) in the Mediterranean region of Türkiye during the years 2021 and 2022. Climate data for the experimental area is presented in **Figure 1**. The average temperature and total precipitation were 20.2 °C and 106.8 mm in 2021 (May-September), 19.7 °C and 122.0 mm in 2022, and 20.2 °C and 140.0 mm for the long-year average (1929-2020), respectively.

Selected physical and chemical properties of the experimental area soils are given in **Tables 1** and **2**.

**Table 1:** Selected physical properties of the experimental area soils.

Soil depth (cm)	Bulk density (g cm^-3^)	Soil structure				Field capacity		Wilting point		Available water capacity (AWC)	
		Clay	Silt	Sand	Class	%	mm	%	mm	%	mm
0-30	1.34	38.57	32.47	28.96	CL	22.25	88.30	10.40	41.82	11.85	46.48
30-60	1.51	38.67	32.91	28.42	CL	21.92	99.15	11.36	51.38	10.56	47.77
60-90	1.3	38.81	34.82	26.37	CL	22.69	90.00	11.38	44.49	11.31	45.51
90-120	1.36	38.75	30.98	30.27	CL	21.56	87.92	11.39	46.45	10.17	41.47
0–90 cm							277.45		137.69		139.76
0–120 cm							365.37		184.14		181.23

**Table 2:** Selected chemical properties of the experimental area soils.

Soil depth (cm)	EC (μS cm^-1^)	pH	CaCO3 (%)	Organic matter (%)	N (%)	P (mg kg^-1^)	K (cmol kg^-1^)
0-30	166	7.99	20.29	1.56	0.079	5.00	1.94
30-60	156	8.05	21.58	0.54	0.031	2.50	0.75
60-90	161	8.04	21.01	0.61	0.039	1.00	0.58
90-120	704	7.34	20.15	0.48	0.031	0.50	0.44

For each plot, soil samples were collected from three representative points and composited for gravimetric soil moisture determination at four soil depths (0-30, 30-60, 60-90, and 90–120 cm). The experimental area soils have a clay-loam (CL) texture and a high water retention capacity (365.37 mm) at a depth of 0–120 cm. Based on the measured EC values (156-704 µS cm^-1^), the soil was classified as non-saline, while the pH values (7.34-8.05) indicated slightly alkaline conditions. The soil was also calcareous (20.15–21.58% CaCO_3_) and low in organic matter and nitrogen.

### Field experiments

In the experiment, the Greengo sorghum x Sudan grass hybrid variety obtained from Ulusoy Seed Company was used. The variety is an annual, has a high leaf/stalk ratio, can reach a plant height of 250–280 cm, has a broad leaves structure, and is suitable for silage production (Anonymous, 2024).

The field experiment was conducted in Randomized Complete Blocks Design (RCBD) with split plots, with 3 replications according to the trial design. In the study, which was planned with nitrogen rates placed in the main plots and irrigation treatments in the subplots, a total of 30 different treatments were examined, consisting of 5 different nitrogen rates and 6 different irrigation levels.

Each subplot consisted of five rows spaced 70 cm apart, with a plot width of 3.5 m and a plot length of 8 m (28 m²). To facilitate field irrigation management, the three replicate subplots associated with each nitrogen rate were arranged consecutively to form 30 main plots. A 1.4 m buffer zone was maintained between adjacent main plots to prevent water and fertilizer movement among treatments. The experiment consisted of 90 subplots arranged as 30 main plots. Based on this field layout, the experimental field measured 145.6 m in width and 24 m in length, resulting in a total experimental area of 3494.4 m².

The seeding process was carried out using a pneumatic seeder with row spacing set at 70 cm and in-row spacing set at 8–10 cm, with the seed placed directly onto the drippers on May 10, 2021, for the first year, and May 13, 2022, for the second year. Triple superphosphate (TSP 42%) fertilizer was used as the phosphorus fertilizer, and, based on soil analysis, 8 kg of phosphorus (P_2_O_5_) per decare was applied entirety during sowing. One-third of the nitrogen fertilizer was applied during sowing, and the remaining amount was applied in two fertigation applications according to the assigned nitrogen treatments.

The harvest process was carried out on September 20, 2021, and September 23, 2022, when the sorghum plant reached the dough stage. Taking edge effects into account during harvesting, one row on the outside and one meter from the beginning and end of the plot were excluded from the assessment, and the remaining part of the plot was harvested.

### Irrigation treatments

Irrigation was carried out using a subsurface drip irrigation system. In the subsurface drip irrigation system, laterals were placed at a depth of 30 cm and a spacing of 70 cm below the soil surface using a burying machine attached to the rear of a tractor.

Six different irrigation treatments were established based on the replenishment percentage of the seven-day cumulative reference evapotranspiration (ETo): 0% (rainfed, I0), 25% (I25), 50% (I50), 75% (I75), 100% (I100), and 125% (I125). Five nitrogen rates (0, 80, 160, 240, and 320 kg N ha^-1^) were applied. In the irrigation treatments, I0 represented non-irrigated conditions under natural rainfall, while I25, I50, and I75 corresponded to the application of 25%, 50%, and 75% of the irrigation water applied in the I100 treatment, respectively. The I100 treatment represent full irrigation based on 100% replenishment of the seven-day cumulative reference evapotranspiration (ETo), whereas I125 received 125% of the irrigation water applied to the I100 treatment.

Before irrigation treatments were initiated, sprinkler irrigation was used to apply the same amount of irrigation water to all plots until the plants reached a length of 15–20 cm to ensure homogeneous emergence and establishment of plants. The irrigation interval in the experiment was set at seven days. The amount of irrigation water was calculated using the following formula, adjusted according to the ratios of the seven days total reference crop water consumption in the irrigation plots. Reference crop water consumption values were obtained from a meteorological station located approximately 500 m away from the trial area, used for agricultural purposes, and calculating reference crop water consumption according to the Penman-Monteith method. Irrigation water quantities were measured with water meters placed at the head of each plot (Equation 1).

(1) $$\text{I }=\text{ A}\times \text{E}{\text{T}}_{0}\times \text{P}\times \text{R}$$

In the equation, I: Irrigation water, liters A: Plot area m^2^, ET_0_: Cumulative reference crop water consumption amount during the irrigation interval, mm, P: Cover percentage, % and R: ET_0_ ratio.

To determine the canopy percentage, the plant crown width was measured and calculated before each irrigation (Equation 2) (Ertek and Kanber, 2000).

(2) $$\text{P}=\;\left(\text{z }{\text{y}}^{-1}\right)\;\times \;100$$

In the equation, z: Plant crown width, cm, y: Row spacing, cm.

After irrigation began, soil moisture content was determined using the gravimetric method by taking disturbed soil samples from soil depths of 0-30, 30-60, 60-90, and 90–120 cm according to the planned irrigation intervals. Considering the effective root depth (0–90 cm), crop water consumption (ET) was calculated using the equation below, based on the water budget approach, from soil samples taken from each 30 cm layer (Equation 3) (James, 1993).

(3) ET= I+ R + Cr − Dp ± Rf ± ΔS

In the equation, ET: Crop water consumption, (mm), I: Applied irrigation water, (mm), R: Effective rainfall, (mm), Dp: Deep percolation, (mm), Cr: Capillary rise, mm, Rf: Surface runoff, (mm), and ΔS: Change in soil water content in the soil profile (mm).

In determining the yield response factor for sorghum, calculations were based on the Stewart model, which utilizes the dimensionless parameters of proportional yield reduction and proportional crop water consumption deficit (Equation 4) (Doorenbos and Kassam, 1979).

(4) $$\text{Ky}=\;[1-\left(\text{Ya Y}{\text{m}}^{-1}\right]\;{\left[1-(\text{ETa ET}{\text{m}}^{-1}\right)]}^{-1}$$

In the equation, Ky: Yield response factor, Ya: Actual yield, (kg da^-1^), Ym: Maximum yield, (kg da^-1^), Eta: Actual water consumption, (mm), Etm: Maximum water consumption, (mm).

During the growing period, the total irrigation water applied and yield values were recorded, and the water use efficiency (WUE) and irrigation water use efficiency (IWUE) for each application were calculated using Equations 5 and 6 by Howell et al. (1990).

(5) $$\text{WUE}=Ey\;ET^{-1}$$

(6) $$\text{IWUE}=(Ey-Eyni)I^{-1}$$

In the equations, WUE: Water use efficiency (t (ha×mm)^-1^), IWUE: Irrigation water use efficiency (t (ha×mm)^-1^), Ey: Yield (kg ha^-1^), Eyni: Yield obtained under non-irrigated conditions (kg ha^-1^), ET: Crop water consumption (mm), I: Irrigation water (mm).

### Morphological observations and yields

Plant height (cm), number of leaves, and stalk thickness (mm) were measured on 10 randomly selected plants from each plot, and mean values were calculated (Şimşeksoy, 2020). To determine stalk diameter, the main stalk of the plants was measured using a caliper. After separating the leaves and stalks of the 10 randomly selected plants from each plot, they were weighed separately to determine their fresh weights. The leaves and stalk weights were calculated by calculating their ratio to determine the leaf/stalk ratio as a percentage (Atalay and Ateş, 2020). All plants harvested from each plot were weighed immediately after harvest to determine green forage yield. The green forage yield obtained from each harvested plot was converted to a hectare basis (t ha^-1^) according to the harvested plot area using the following Equation 7 (Akbay, 2022).

(7) $$\text{Green forage yield}=\left(\text{Green forage weight }\left(\text{kg}\right)\times 10000\right)\;{\left(\text{Harvest area }\left(\text{m}2\right)\;\times \;1000\right)}^{-1}$$

The dry matter content was determined by drying approximately 500 g of green forage samples taken from each plot at 70 °C until a constant weight was reached. Dry matter yield (t ha^-1^) was determined by multiplying the green forage yield by the dry matter content. Crude protein yield (t ha^-1^) was obtained by multiplying the dry matter yield by the crude protein content.

### Forage quality and mineral analysis

The dried forage samples were ground using a mill with a 1 mm sieve to prepare them for subsequent chemical analysis. Total nitrogen content was determined using the Kjeldahl method, and crude protein (CP) content was calculated by multiplying nitrogen concentration by 6.25 according to AOAC (1990). Analyses for ADF and NDF were conducted in accordance with the ANKOM Tech Methods (2017). The crude ash content of the sorghum samples was determined by incinerating the samples in an oven at 550 °C for 8 hours (AOAC, 1990).

According to the method described by Jackson (1958), samples were incinerated, extracted with filter paper, and their K, Ca, and Mg contents were determined in ppm using an Atomic Absorption Spectrophotometer. Phosphorus was extracted using the method developed by Olsen et al. (1954) and determined in the Spectrophotometer using the molybdenum phosphoric blue color method.

### Physiological measurements

Leaf greenness (SPAD value) was measured weekly after irrigation began using a portable chlorophyll meter (Chlorophyll meter SPAD-502 Minolta Japan) by taking samples from the area between the leaves vein and leaves margin of the middle leaves of 10 plants marked in the plot before irrigation (Tunalı, 2012). Stomatal conductance was measured using a Delta-T AP4 portable porometer 4–5 weeks after irrigation began, every 15 days before irrigation on 3 plants with mature leaves (Jarvis, 1976).

### Statistical analysis

The experiments were arranged in a Randomized Complete Block Design with split plots and included three replications. Nitrogen application rates were applied to the main plots, whereas irrigation levels were assigned to the sub-plots. The experimental data were analyzed using analysis of variance (ANOVA) with the SAS software package (SAS Institute Inc, 1999). Significant differences among means were then evaluated using the Duncan test for comparisons.

## Results

### Crop water consumption and irrigation

Irrigation water amount, evapotranspiration (ET), forage yield, water use efficiency (WUE), and irrigation water use efficiency (IWUE) values for the experimental years are presented in **Table 3**. Higher irrigation levels resulted in greater irrigation water application and seasonal evapotranspiration in both years. Green forage yield also increased with increasing irrigation levels, although yield increments became less pronounced under the highest irrigation treatment (I125).

**Table 3:** ET, WUE, and IWUE values determined in the study for 2021 and 2022.

Treatments		Irrigation (mm)	ET (mm)	Yield (t ha^-1^)	WUE (t ha^-1^ mm^-1^)	IWUE (t ha^-1^ mm^-1^)
2021	I_0_	259.7	394.2	51.2	0.129	
	I_25_	346.2	468.9	65.4	0.139	0.041
	I_50_	433.0	543.5	83.0	0.153	0.074
	I_75_	519.6	620.2	94.8	0.153	0.084
	I_100_	606.1	693.5	103.0	0.148	0.085
	I_125_	692.6	764.6	104.1	0.136	0.076
2022	I_0_	274.8	464.0	44.7	0.096	
	I_25_	352.9	528.3	59.7	0.113	0.043
	I_50_	430.9	589.8	71.9	0.122	0.063
	I_75_	508.9	657.7	83.4	0.127	0.076
	I_100_	586.9	727.4	91.1	0.125	0.079
	I_125_	664.9	795.6	96.9	0.122	0.079

I0, I25, I50, I75, I100, and I125 represent irrigation treatments corresponding to 0%, 25%, 50%, 75%, 100%, and 125% replenishment of cumulative reference evapotranspiration (ETo), respectively. ET, evapotranspiration; WUE, water use efficiency; IWUE, irrigation water use efficiency.

Water use efficiency and irrigation water use efficiency varied according to irrigation level and experimental year. In general, WUE values were higher under moderate irrigation treatments (I50-I75), whereas IWUE values were highest under I75-I100 treatments, indicating improved irrigation efficiency under moderate-to-full irrigation conditions (**Table 3**).

**Figure 2:**
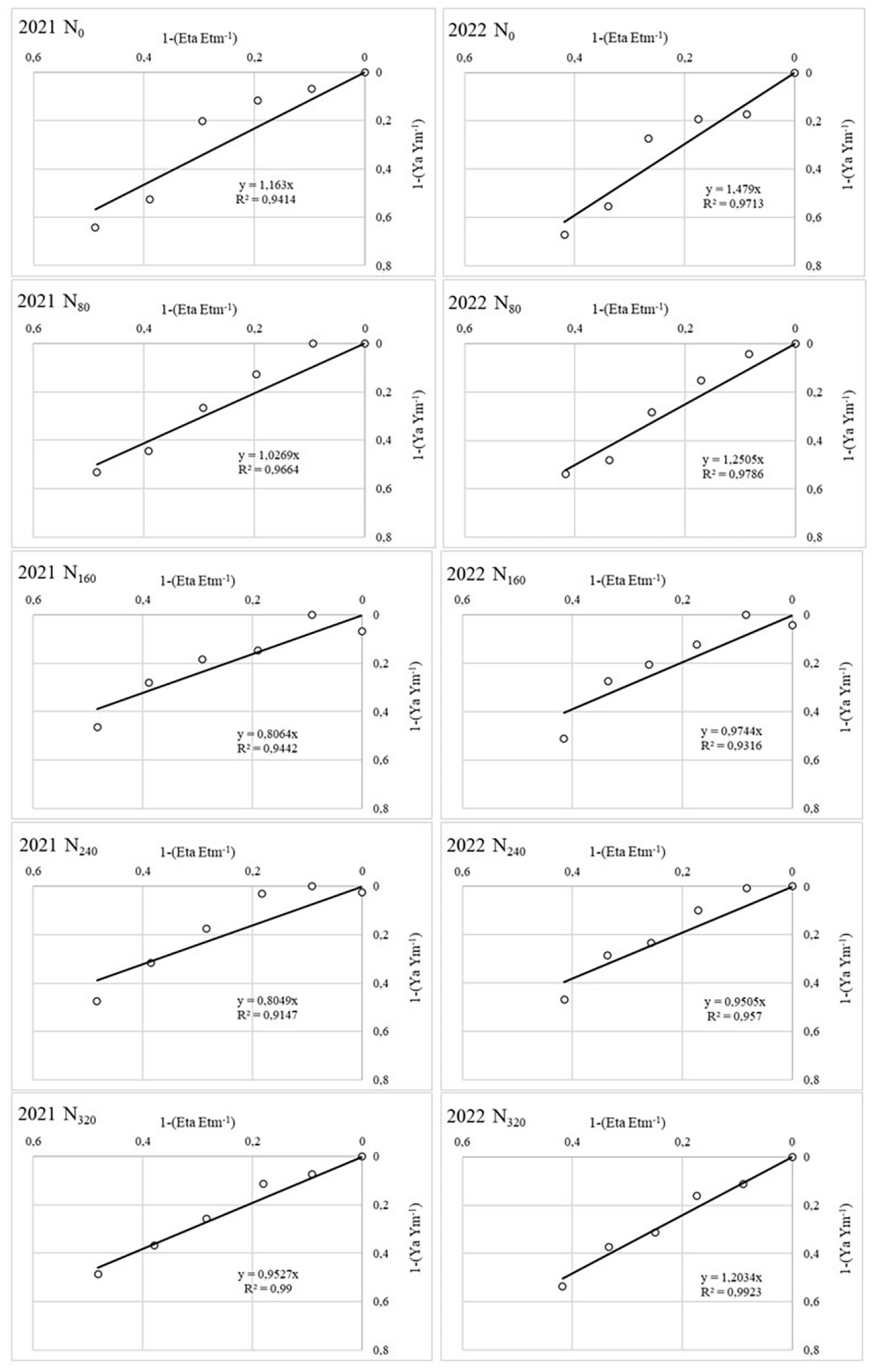
Yield response factor (Ky) for 2021 and 2022.

The yield response factor varied depending on nitrogen rate and experimental year (**Figure 2**). Nitrogen treatments with Ky values greater than 1 showed greater sensitivity to water deficit, whereas treatments with values below 1 were relatively more tolerant. In both years, the N160 and N240 treatments generally exhibited lower Ky values, indicating improved tolerance to water deficit conditions compared with low nitrogen applications.

### Yields and morphological observations

**Figure 3:**
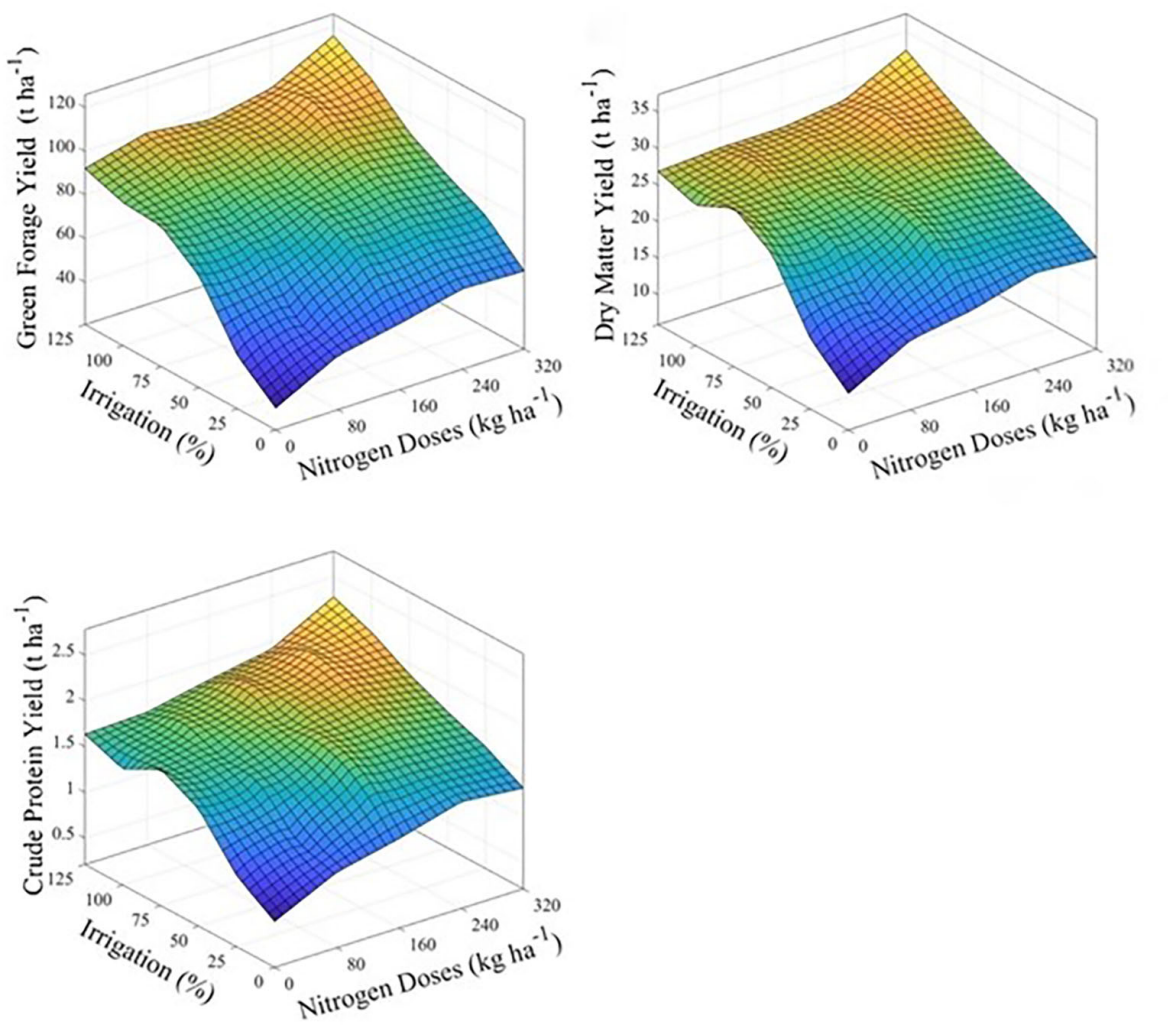
Effects of irrigation level and nitrogen rate on green forage yield, dry matter yield, and crude protein yield of sorghum. Values represent two-year averages.

**Figure 4:**
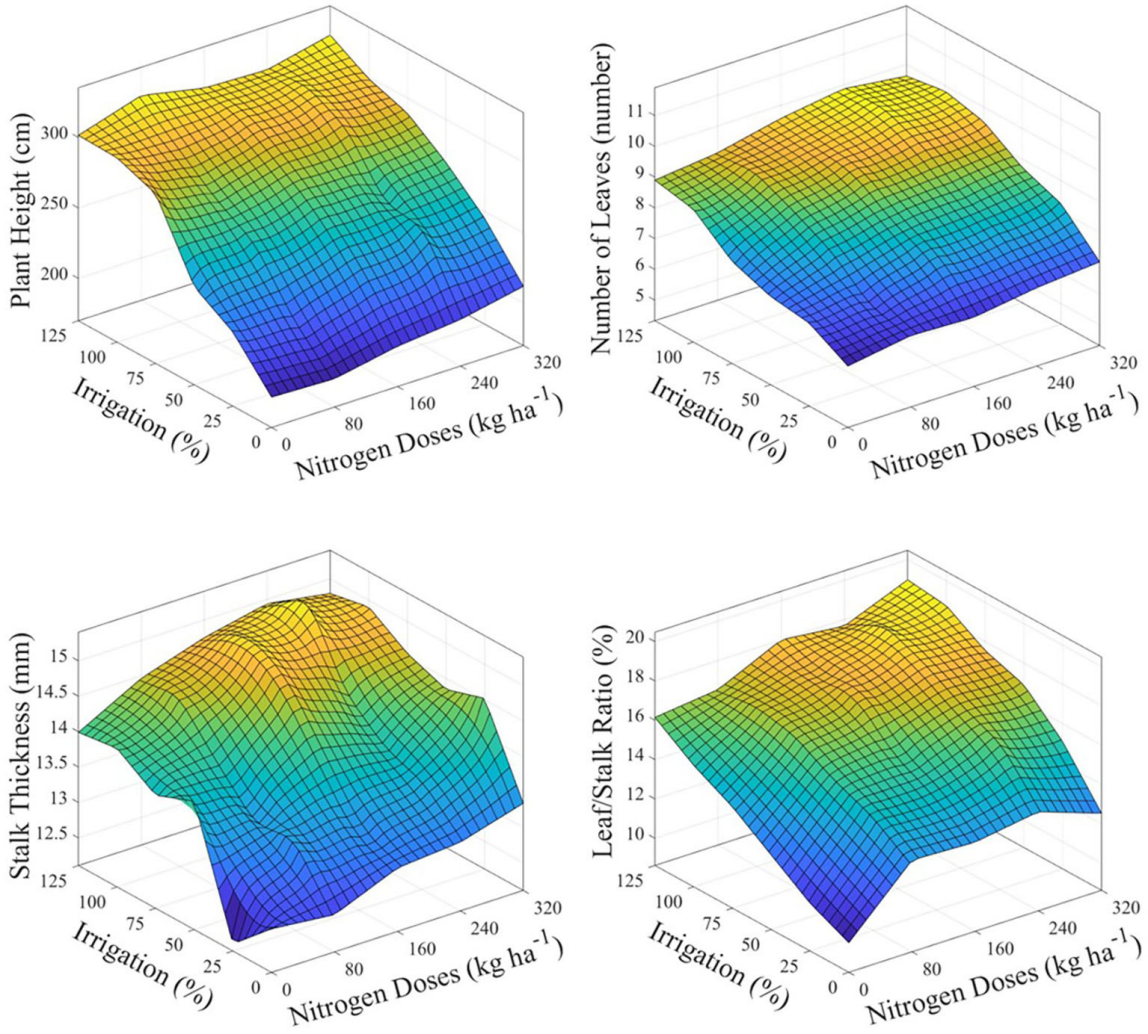
Effects of irrigation level and nitrogen rate on morphological traits of sorghum. Values represent two-year averages.

According to the two-year combined analysis of variance, nitrogen rate, irrigation level, and their interaction significantly affected all yield and morphological parameters (p < 0.01) (**Supplementary Table 1**). Higher irrigation and nitrogen levels improved plant height, number of leaves, stalk diameter, leaf/stalk ratio, green forage yield, dry matter yield, and crude protein yield. The strongest responses were generally observed under I100-I125 irrigation combined with 240–320 kg N ha^-1^ applications (**Figures 3**, **4**; **Supplementary Table 2**). In particular, green forage and dry matter yields markedly increased under higher irrigation and nitrogen combinations, indicating a strong interactive effect of water and nitrogen management on sorghum productivity.

### Forage quality

**Figure 5:**
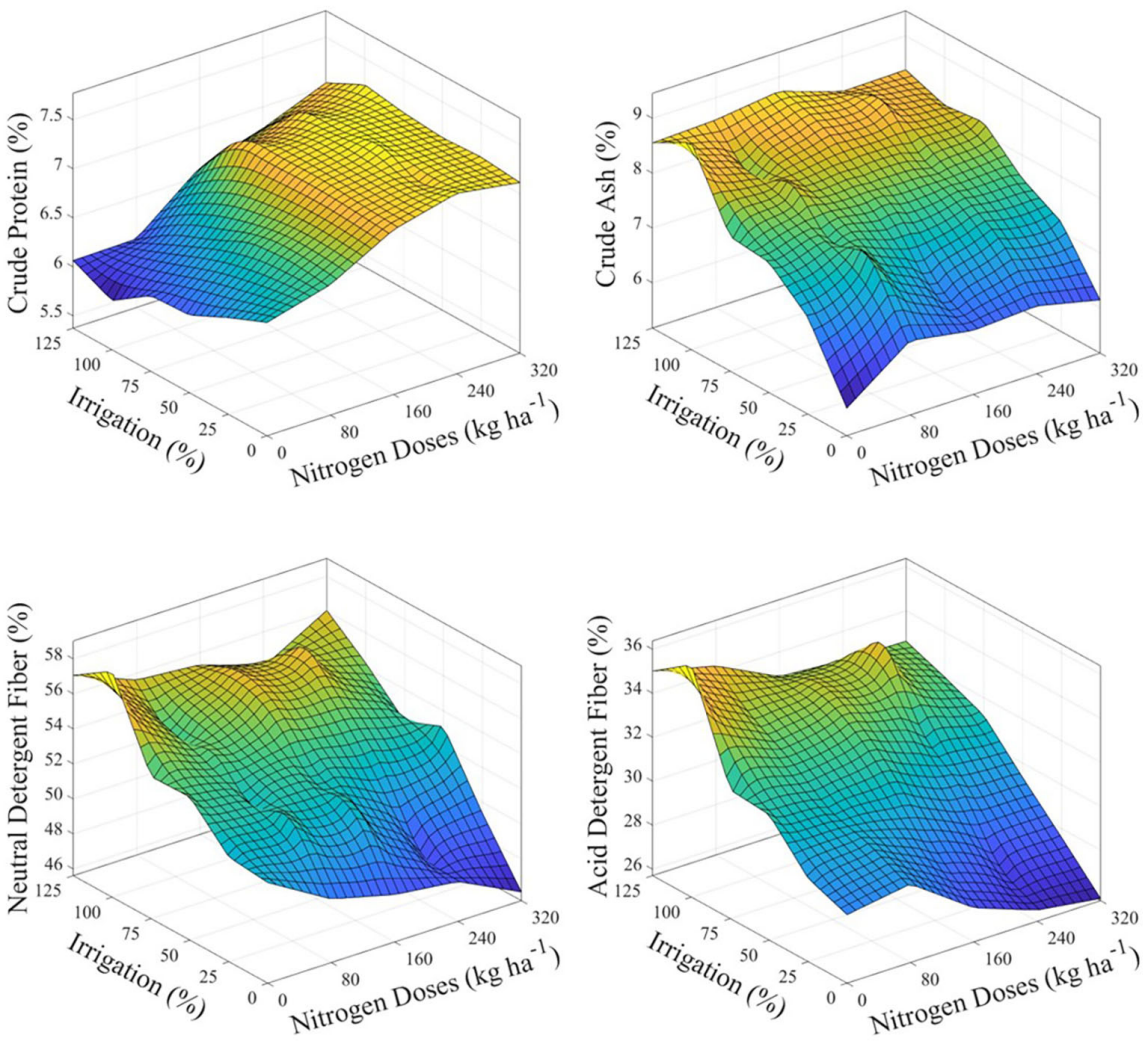
Effects of irrigation level and nitrogen rate on crude protein, crude ash, neutral detergent fiber (NDF), and acid detergent fiber (ADF) contents of sorghum. Values represent two-year averages.

Nitrogen rate, irrigation level, and their interaction significantly affected CP, NDF, and ADF contents (p < 0.01) (**Supplementary Table 1**). Increasing nitrogen applications generally increased CP content, whereas increasing irrigation levels reduced CP values. In contrast, NDF and ADF contents showed an opposite response to nitrogen and irrigation treatments, with higher fiber concentrations generally observed under high irrigation and low nitrogen conditions (**Figure 5**; **Supplementary Table 2**).

The effect of nitrogen rates on crude ash content was not statistically significant, whereas irrigation levels significantly affected crude ash values. Crude ash content generally increased with increasing irrigation levels (**Figure 5**).

### Mineral contents of forage

**Figure 6:**
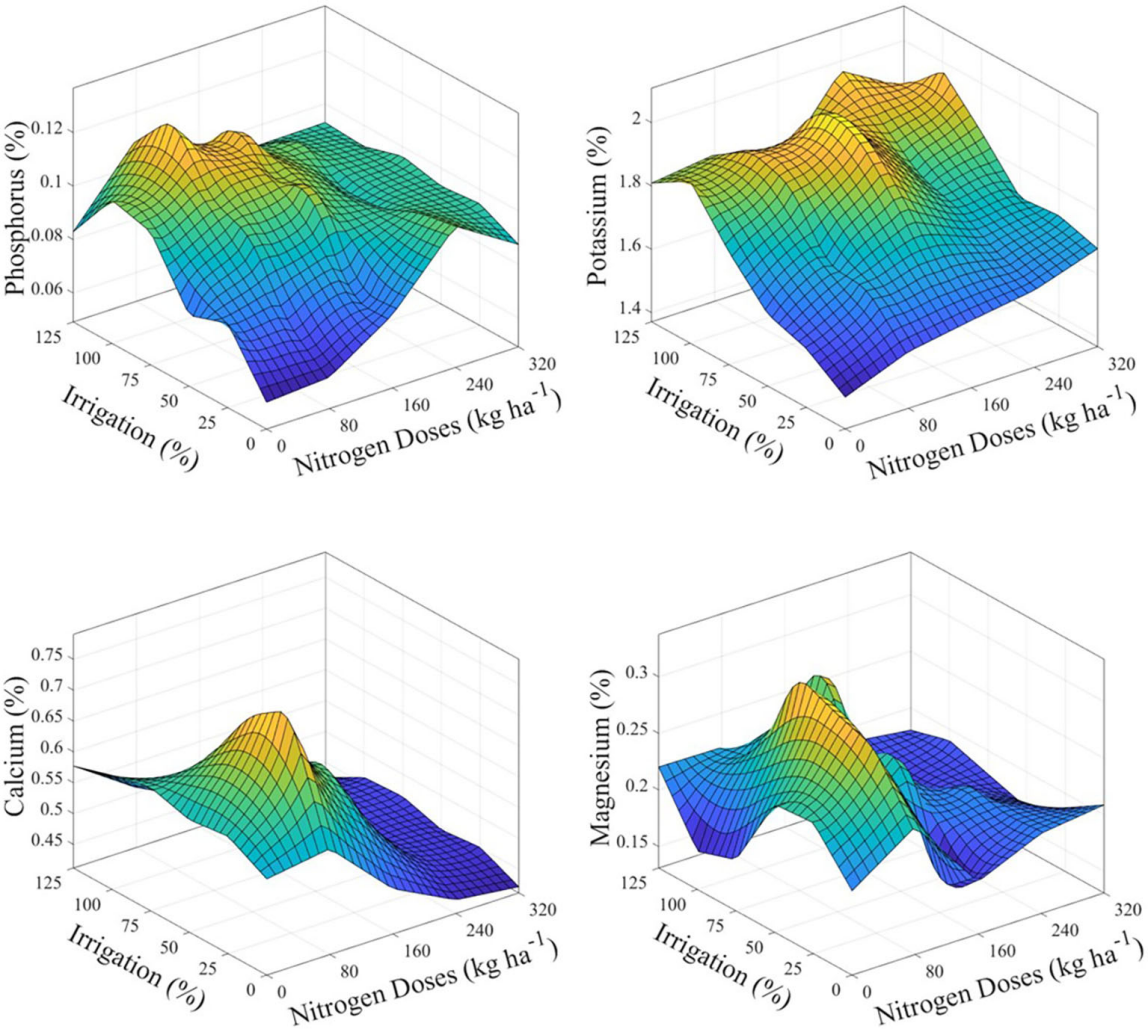
Effects of irrigation level and nitrogen rate on mineral composition of sorghum forage. Values represent two-year averages.

Nitrogen rate, irrigation level, and their interaction significantly affected phosphorus (P), potassium (K), calcium (Ca), and magnesium (Mg) contents of sorghum forage (p < 0.01) (**Supplementary Table 1**). Increasing irrigation levels generally increased P, K, and Mg concentrations, whereas Ca content decreased under higher irrigation conditions. Similarly, increasing nitrogen rates increased P and K contents but tended to reduce Ca and Mg concentrations (**Figure 6**; **Supplementary Table 2**).

### Physiological measurements

**Figure 7:**
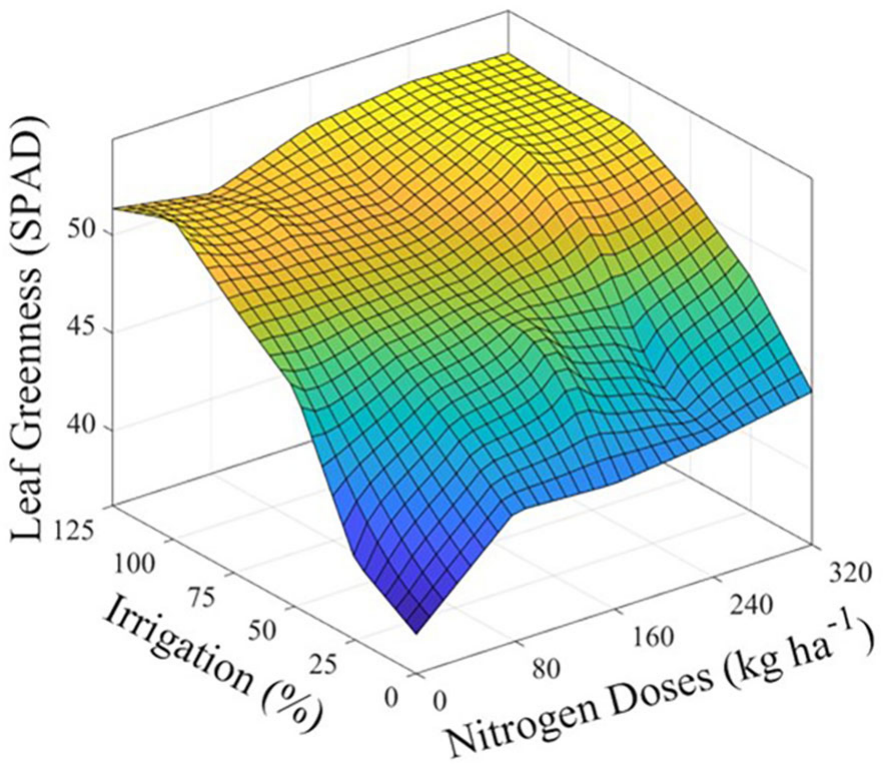
Effects of irrigation level and nitrogen rate on leaf greenness (SPAD) values of sorghum. Values represent two-year averages.

Nitrogen rate, irrigation level, and their interaction significantly affected leaf greenness (SPAD) and stomatal conductance values (p < 0.01) (**Supplementary Table 1**). Leaf greenness increased with increasing irrigation and nitrogen applications, with the strongest responses generally observed under I100-I125 irrigation combined with 240–320 kg N ha^-1^ treatments (**Figure 7**; **Supplementary Table 3**).

**Figure 8:**
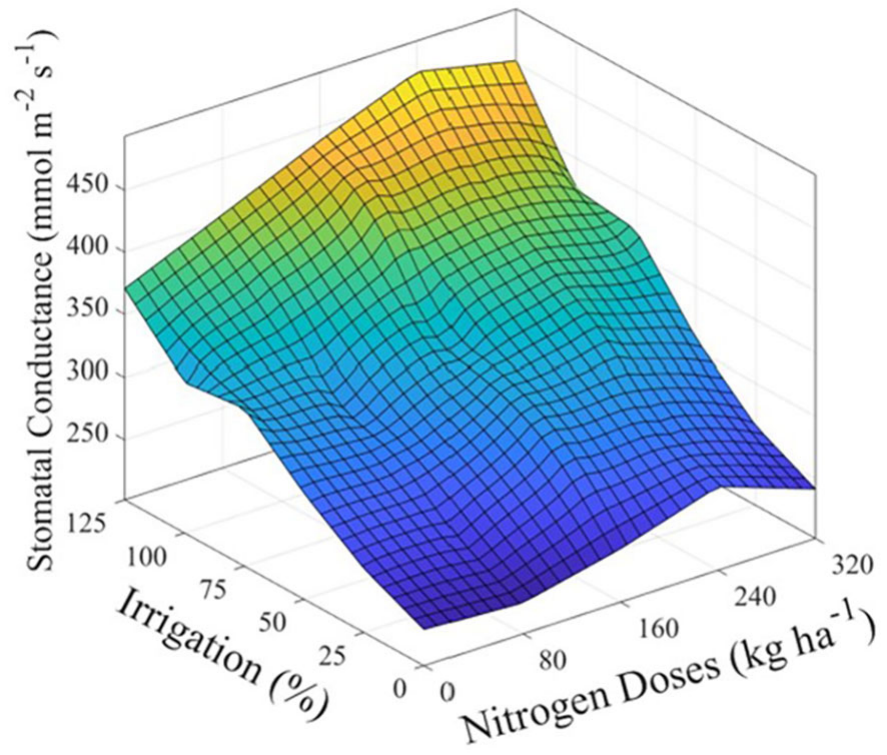
Effects of irrigation level and nitrogen rate on stomatal conductance of sorghum. Values represent two-year averages.

Similarly, stomatal conductance increased under higher irrigation and nitrogen levels, whereas the lowest values were observed under rainfed and non-fertilized conditions (**Figure 8**; **Supplementary Table 3**).

## Discussion

### Crop water consumption and irrigation

The present study revealed a positive relationship between irrigation level and seasonal crop water consumption (**Table 3**). Seasonal evapotranspiration (ET) values ranged from 394.2 to 795.6 mm during the approximately 130–135 day sorghum growing period, depending on irrigation treatment and year. Similar trends have been reported in previous studies conducted under different irrigation systems and environmental conditions (Dündar, 2019; Kaplan et al., 2019; Durusoy, 2022; Sündük, 2023). However, the magnitude of seasonal water consumption varied considerably among studies. These differences may be attributed to variations in climatic conditions, soil characteristics, irrigation methods, growing season length, and sorghum genotype. For example, higher temperatures and evaporative demand generally increase crop water use, whereas soils with greater water retention capacity may buffer short-term water deficits and alter evapotranspiration dynamics.

Water use efficiency (WUE) and irrigation water use efficiency (IWUE) also varied according to irrigation treatment (**Table 3**). The highest WUE values were generally obtained under moderate irrigation levels (I50-I75), indicating that sorghum was able to maintain relatively high biomass production while using less water. Similar responses have been reported by Dündar (2019) and Kaplan et al. (2019). Although the WUE values observed in the present study were generally within the range reported in the literature, some differences in IWUE values were observed. These discrepancies are likely related to differences in irrigation scheduling, environmental conditions, soil water storage capacity, and yield potential among experimental sites. The results suggest that moderate deficit irrigation may improve both water use efficiency (WUE) and irrigation water use efficiency (IWUE) while maintaining acceptable forage yield, which is particularly important for sustainable forage production under conditions of limited water availability. From a practical perspective, irrigation levels between I75 and I100 provided a favorable balance between forage yield, WUE, and IWUE under semi-arid conditions.

The yield response factor provides an indication of crop sensitivity to water deficit during the growing season (**Figure 2**). According to FAO (2023), sorghum generally exhibits a Ky value close to 0.90 under normal growing conditions, reflecting its relatively high drought tolerance. In the present study, Ky values varied according to nitrogen rate, with N160 and N240 treatments generally exhibiting lower Ky values than low-nitrogen treatments. This finding suggests that adequate nitrogen nutrition may improve the ability of sorghum to maintain productivity under water deficit conditions. Similar observations have been reported by Aydinşakir et al. (2018); Kaplan et al. (2019), and Moray (2020), the substantial variation among studies has also been documented.

The timing of water deficit is also an important factor influencing sorghum performance. Sorghum can tolerate moderate water deficits during vegetative growth better than many cereal crops; however, water limitation during stem elongation, panicle initiation, and reproductive stages may substantially reduce biomass accumulation and yield. Therefore, maintaining adequate soil moisture during critical growth periods appears to be essential for maximizing both forage yield and water use efficiency under subsurface drip irrigation conditions.

### Yields and morphological observations

In the present study, increasing irrigation and nitrogen levels significantly improved green forage yield, dry matter yield, and crude protein yield (**Supplementary Table 2**; **Figures 3**, **4**). The positive response of yield to irrigation was expected, as adequate water availability supports cell expansion, nutrient transport, photosynthetic activity, and biomass accumulation. Similarly, nitrogen promotes vegetative growth and protein synthesis, leading to increased forage production and nutritive value. The significant nitrogen x irrigation interaction observed in this study indicates that the effectiveness of nitrogen fertilization depends largely on sufficient water availability, as nutrient uptake and utilization are restricted under water-deficit conditions.

Similar findings have been reported by Cosentino et al. (2012); Dahmardeh et al. (2015); Kaplan et al. (2019), and Varol et al. (2025), who reported that increasing irrigation and nitrogen levels generally enhanced forage yield in sorghum. Water deficit has been shown to reduce biomass accumulation by limiting photosynthesis, leaves expansion, and dry matter production, thereby decreasing both dry matter and crude protein yields (Carmi et al., 2005; Gönülal, 2020). Likewise, increasing nitrogen availability has consistently been associated with higher crude protein yield due to its direct role in protein synthesis and dry matter production (Yüksel, 2006; Atalay and Ateş, 2020).

Plant height, which is considered an important indicator of plant response to water deficit, increased significantly with increasing irrigation and nitrogen levels (**Supplementary Table 2**). Adequate water supply maintains cell turgor and stem elongation, whereas nitrogen supports vigorous vegetative growth through enhanced chlorophyll formation and photosynthetic activity. Similar responses have been reported by Mahinda (2014); Abd El-Mageed et al. (2018); Yüksel (2006); Khaled (2013), and Sadighfard and Geren (2022).

The number of leaves and stalk diameter were also positively affected by irrigation and nitrogen applications (**Supplementary Table 2**). Water deficit generally limits leaves initiation and expansion, reducing the photosynthetic surface area available for biomass production. In contrast, adequate irrigation and nitrogen supply promote canopy development and stem growth. Similar trends have been reported by Sanchez et al. (2002); Rostampour (2013); Mahinda (2014); Aydinşakir et al. (2018); Yüksel (2006); Çoban (2018); Rouhbakhsh (2013); El-Samnoudi et al. (2019); Akbay (2022), and Atalay and Ateş (2020).

The leaf/stalk ratio is an important indicator of forage quality because leaves generally contain higher concentrations of digestible nutrients and lower concentrations of structural carbohydrates than stalk tissues. Therefore, an increase in the leaf/stalk ratio may contribute positively to forage intake and digestibility (Hatfield, 1993). In the present study, both irrigation and nitrogen management significantly influenced this parameter (**Supplementary Table 2**). Previous studies have reported variable responses of leaf/stalk ratio to irrigation practices (Kaplan et al., 2019; Dündar et al., 2020), suggesting that the response may depend on cultivar characteristics, environmental conditions, and the balance between leaf/stem ratio under different management practices.

### Forage quality

In the present study, increasing irrigation levels decreased crude protein (CP) content, whereas increasing nitrogen rates increased CP content (**Supplementary Table 2**; **Figure 5**). The reduction in CP concentration under higher irrigation levels may be attributed to a dilution effect caused by increased biomass production, while the positive effect of nitrogen fertilization is directly related to its role in amino acid and protein synthesis. Similar responses have been reported by Kaplan et al. (2019), Dündar et al. (2020), Varol et al. (2025), and Atalay and Ateş (2020). The significant interaction between irrigation and nitrogen treatments further suggests that forage protein concentration is strongly influenced by the balance between biomass accumulation and nitrogen availability.

Neutral detergent fiber and acid detergent fiber contents exhibited trends opposite to those observed for CP (**Supplementary Table 2**; **Figure 5**). Increasing irrigation levels generally increased NDF and ADF concentrations, whereas increasing nitrogen rates reduced both fiber fractions. Since NDF is negatively associated with voluntary feed intake and ADF is negatively associated with digestibility, these changes have important implications for forage quality and animal performance (Yang and Beauchemin, 2009). The increase in fiber content under higher irrigation levels may be associated with enhanced stem growth and lignification, whereas nitrogen fertilization promotes the development of more digestible plant tissues. Similar observations have been reported by Newman (2014); Amaducci et al. (2000), and Kilcer et al. (2002). These findings indicate that irrigation and nitrogen management influence not only forage yield but also the nutritional value of sorghum forage.

Crude ash content was significantly affected by irrigation treatments but not by nitrogen rates (**Supplementary Table 2**). The crude ash values obtained in the present study were generally within the ranges reported for sorghum under different environmental conditions and management practices (Aydınoğlu et al., 2007; Kumari et al., 2013; Chakravarthi et al., 2017; Akbay, 2022; Chalil, 2023). The increase in crude ash content with increasing irrigation levels could be associated with improved mineral uptake and translocation resulting from greater soil water availability.

### Mineral contents of forage

Irrigation level, nitrogen rate, and their interaction significantly affected the mineral composition of sorghum forage (**Supplementary Table 2**; **Figure 6**). Increasing irrigation levels generally increased phosphorus (P), potassium (K), and magnesium (Mg) concentrations, whereas calcium (Ca) concentration decreased. Similarly, increasing nitrogen rates increased P and K contents while reducing Ca and Mg concentrations.

The increase in P and K concentrations under higher irrigation levels could be associated with improved nutrient mobility in the soil and enhanced nutrient uptake resulting from greater root activity under favorable moisture conditions. Potassium plays a key role in osmotic regulation and stomatal function, while phosphorus is essential for energy transfer and plant growth. Therefore, improved water availability may facilitate the acquisition and translocation of these nutrients within the plant. The P and K values obtained in the present study were generally within or above the ranges reported by previous studies conducted under different environmental conditions (Aydınoğlu, 2005; Gökkuş et al., 2017; Uzun et al., 2017; Kaplan, 2021; Emmanuel et al., 2022). Furthermore, K concentrations exceeded the minimum requirements reported by National Research Council (1984) and Tejada et al. (1985), indicating adequate potassium nutrition for livestock feeding purposes.

In contrast, Ca and Mg concentrations generally declined with increasing irrigation and nitrogen levels. This response may be related to a dilution effect associated with increased biomass production, as well as possible antagonistic interactions among mineral nutrients during uptake and translocation. Similar trends have been reported in previous studies evaluating mineral composition in sorghum and other forage crops (Uzun et al., 2017; Kaplan, 2021). Nevertheless, the Ca and Mg concentrations determined in the present study remained within acceptable ranges for animal nutrition and were generally consistent with values reported in the previous studies (Aydınoğlu, 2005; Gökkuş et al., 2017; Emmanuel et al., 2022).

Overall, the results demonstrate that water and nitrogen management affect not only forage yield and quality but also mineral nutrition. These changes should be considered when developing fertilization and irrigation strategies aimed at optimizing both forage productivity and nutritional value under semi-arid conditions.

### Physiological measurements

In the present study, leaf greenness (SPAD) and stomatal conductance increased with increasing irrigation and nitrogen applications (**Supplementary Table 3**; **Figures 7**, **8**). Water deficit is known to reduce chlorophyll synthesis and accelerate chlorophyll degradation, thereby limiting photosynthetic activity and plant growth (Abdalla and Abdelwahab, 1995; Sangakkara et al., 1996). Similarly, Kabay and Şensoy (2016) reported that unfavorable environmental conditions reduce chlorophyll status, resulting in lower yield and forage quality. The higher SPAD values observed under increased irrigation and nitrogen levels in the present study suggest improved nitrogen availability and photosynthetic capacity. Since leaves nitrogen content is closely associated with chlorophyll formation, the positive response of SPAD values to nitrogen fertilization is consistent with previous findings reported by Ullah et al. (2015) and Ajeigbe et al. (2018).

Stomatal conductance was also significantly influenced by irrigation and nitrogen management (**Supplementary Table 3**; **Figure 8**). The higher stomatal conductance values observed under well-watered treatments indicate that plants maintained greater gas exchange capacity and photosynthetic activity under favorable moisture conditions. In contrast, the reduction in stomatal conductance under water-deficit treatments suggests a physiological response aimed at limiting transpirational water loss. Similar reductions in stomatal conductance under drought stress have been reported by De Souza et al. (2015) and Allamine et al. (2023).

Stomatal regulation is one of the primary mechanisms by which sorghum adapts to water deficit environments. When soil moisture declines, partial stomatal closure reduces water loss and improves short-term water conservation; however, prolonged reductions in stomatal conductance may also restrict carbon assimilation and biomass accumulation (Chaves et al., 2002; Osakabe et al., 2014). Therefore, the higher stomatal conductance values observed under I100 and I125 treatments likely contributed to the greater forage yields recorded in these treatments. These results indicate that adequate irrigation and nitrogen supply help maintain physiological activity and support productive growth in sorghum under semi-arid conditions.

Plants may cope with water deficit through both avoidance and tolerance mechanisms. Water-stress avoidance is associated with traits such as root system expansion and effective stomatal regulation (Trejo and Davies, 1991; Barradas et al., 1994), whereas tolerance mechanisms involve osmotic adjustment and membrane protection (Mullet and Whitsitt, 1996). The physiological responses observed in the present study suggest that sorghum employed stomatal regulation as an important adaptive strategy under reduced irrigation levels, contributing to its well-known resilience to moderate drought conditions.

## Conclusion

This two-year study demonstrated that irrigation and nitrogen management significantly affected forage yield, quality characteristics, water use efficiency, mineral composition, and physiological responses of sorghum grown under subsurface drip irrigation conditions. Increasing irrigation and nitrogen levels generally improved biomass production, crude protein yield, leaf greenness (SPAD), and stomatal conductance, whereas water deficit negatively affected plant growth and physiological performance. The results also demonstrated a strong interaction between irrigation and nitrogen management, indicating that the benefits of nitrogen fertilization were maximized under adequate water supply. Balanced nitrogen applications improved forage quality by reducing NDF and ADF concentrations while increasing crude protein content.

Although the highest values for some individual traits were obtained under I125 irrigation and 320 kg N ha^-^¹, these treatments did not provide agronomic benefits proportional to the additional water and nitrogen inputs. In contrast, full irrigation (I100) combined with 240 kg N ha^-^¹ provided a more favorable balance among forage yield, quality, physiological performance, and irrigation water use efficiency. Therefore, this treatment is recommended as the optimum management strategy for silage sorghum production. Where reducing fertilizer input is a priority, 160 kg N ha^-^¹ may also be considered a practical alternative, as it maintained satisfactory overall performance while requiring substantially less nitrogen input.

Overall, the study highlights the potential of subsurface drip irrigation as an effective approach for improving resource use efficiency in forage sorghum cultivation under semi-arid environments. Future studies should further investigate long-term soil effects, nitrogen use efficiency, and the economic performance of different irrigation fertilization strategies under varying climatic conditions.

## Data Availability

The original contributions presented in the study are included in the article/**Supplementary Material**. Further inquiries can be directed to the corresponding author.
