# Supplementary material for: Interactive effects of nitrogen rates and deficit irrigation on yield, forage quality, and water productivity of silage sorghum under subsurface drip irrigation
Source: Front Plant Sci. 2026 Jul 29;17:1832758. doi: 10.3389/fpls.2026.1832758 (PMC13462259; doi:10.3389/fpls.2026.1832758)
Supplement: Supplementary file 1 [file Table1.docx]

Supplemental Table 1. Results of analysis of variance of the traits determined.

| **Sources   of  Variations** | **df** | | **Green  Forage  Yield** | | **Hay  Yield** | | **Crude  Protein  Yield** | | **Plant  Height** | | **Number  of  Leaf** | | **Stalk  Thickness** | | **Leaf/Stalk  Ratio** | **Crude  Protein  Ratio** | | **Crude  Ash** |  |
| --- | --- | --- | --- | --- | --- | --- | --- | --- | --- | --- | --- | --- | --- | --- | --- | --- | --- | --- | --- |
| **Block (year)** | 4 | | ns | | ns | | ns | | ns | | ns | | ns | | ns | ns | | ns |  |
| **Year** | 1 | | ** | | ** | | ** | | ** | | ** | | ** | | ** | ** | | ** |  |
| **Nitrogen (N)** | 4 | | ** | | ** | | ** | | ** | | ** | | ** | | ** | ** | |  |  |
| **N x Year** | 4 | | ns | | ns | | ns | | ns | | ** | | ns | | ** | ** | | ** |  |
| **Error 1** | 16 | |  | |  | |  | |  | |  | |  | |  |  | |  |  |
| **Irrigation Level (IL)** | 5 | | ** | | ** | | ** | | ** | | ** | | ** | | ** | ** | | ** |  |
| **IL x Year** | 5 | | ** | | ** | | ** | | ** | | ** | | ** | | ** | ** | | ** |  |
| **N x IL intr.** | 20 | | ** | | ** | | ** | | ** | | ** | | ** | | ** | ** | | ** |  |
| **N x IL x Year intr.** | 20 | | ns | | ns | | ns | | ** | | ns | | ** | | ** | ** | | ** |  |
| **Error 2** | 100 | |  | |  | |  | |  | |  | |  | |  |  | |  |  |
| **Corrected Total** | 179 | |  | |  | |  | |  | |  | |  | |  |  | |  |  |
| **Coeff Var** |  | | 5.08 | | 5.70 | | 6.10 | | 1.18 | | 3.75 | | 2.30 | | 3.67 | 2.04 | | 3.18 |  |
|  |  |  | |  | |  | |  | |  | |  | |  | | |  | | |
|  |  | **NDF** | | **ADF** | | **P** | | **K** | | **Ca** | | **Mg** | | **Leaf greenness (SPAD)** | | | **Stomatal  Conductance** | | |
| **Block (year)** | 4 | ns | | ns | | ns | | ns | | ns | | ns | | ns | | | ns | | |
| **Year** | 1 | ** | | ** | | ns | | ns | | ** | | ** | | * | | | ** | | |
| **Nitrogen (N)** | 4 | ** | | ** | | ** | | ** | | ** | | ** | | ** | | | ** | | |
| **N x Year** | 4 | ** | | ** | | ** | | ns | | ** | | ns | | ns | | | ** | | |
| **Error 1** | 16 |  | |  | |  | |  | |  | |  | |  | | |  | | |
| **Irrigation Level (IL)** | 5 | ** | | ** | | ** | | ** | | ** | | ** | | ** | | | ** | | |
| **IL x Year** | 5 | ** | | ** | | ** | | ** | | ** | | ** | | ns | | | ** | | |
| **N x IL intr.** | 20 | ** | | ** | | ** | | ** | | ** | | ** | | ** | | | ** | | |
| **N x IL x Year intr.** | 20 | ** | | ** | | ns | | ** | | ** | | ns | | ns | | | ** | | |
| **Error 2** | 100 |  | |  | |  | |  | |  | |  | |  | | |  | | |
| **Corrected Total** | 179 |  | |  | |  | |  | |  | |  | |  | | |  | | |
| **Coeff Var** |  | 1.62 | | 1.17 | | 2.97 | | 3.32 | | 3.21 | | 4.88 | | 1.65 | | | 4.40 | | |

Supplemental Table 2. Effects of nitrogen doses and irrigation practices on yield, morphological characteristics, quality, and mineral contents in sorghum. (The values in table are presented as the average of 2-year research results)

| **Irrigation Level** | **Green Forage Yield (t ha^-1^)** | | | | |  | **Irrigation Level** | **Hay Yield (t ha^-1^)** | | | | |  | **Irrigation Level** | **Crude Protein Yield (t ha^-1^)** | | | | |
| --- | --- | --- | --- | --- | --- | --- | --- | --- | --- | --- | --- | --- | --- | --- | --- | --- | --- | --- | --- |
|  | **Fertilizer Doses** | | | | |  |  | **Fertilizer Doses** | | | | |  |  | **Fertilizer Doses** | | | | |
|  | **N_0_** | **N_80_** | **N_160_** | **N_240_** | **N_320_** |  |  | **N_0_** | **N_80_** | **N_160_** | **N_240_** | **N_320_** |  |  | **N_0_** | **N_80_** | **N_160_** | **N_240_** | **N_320_** |
| **I_0_** | 31.91 l | 45.06 k | 51.37 ıj | 55.77 ı | 55.56 ı |  | **I_0_** | 11.31 k | 15.17 k | 16.83 jk | 18.48 j | 17.96 j |  | **I_0_** | 0.73 n | 1.03 lm | 1.18 kl | 1.34 jk | 1.27 jk |
| **I_25_** | 42.80 k | 52.08 ıj | 72.50 h | 73.89 gh | 71.44 h |  | **I_25_** | 14.71 k | 17.21 jk | 23.84 g-ı | 22.74 hı | 21.79 ı |  | **I_25_** | 0.93 m | 1.16 kl | 1.69 d-h | 1.63 d-ı | 1.56 g-ı |
| **I_50_** | 70.89 h | 70.36 h | 80.77 fg | 84.30 f | 81.07 fg |  | **I_50_** | 23.72 g-ı | 23.00 hı | 24.97 e-h | 25.23 e-h | 24.11 g-ı |  | **I_50_** | 1.47 ıj | 1.54 hı | 1.78 d-f | 1.83 cd | 1.73 d-h |
| **I_75_** | 78.57 f-h | 83.45 f | 86.58 ef | 99.05 b-d | 97.86 b-d |  | **I_75_** | 25.44 d-h | 26.08 c-g | 25.35 e-h | 28.84 b | 28.52 bv |  | **I_75_** | 1.57 f-ı | 1.70 d-h | 1.80 de | 2.08 ab | 2.06 ab |
| **I_100_** | 81.91 fg | 94.91 cd | 100.24 b-d | 105.30 b | 102.86 bc |  | **I_100_** | 24.23 f-ı | 28.08 b-d | 29.29 b | 29.70 ab | 28.85 b |  | **I_100_** | 1.44 ıj | 1.77 d-g | 2.04 b | 2.15 ab | 2.08 ab |
| **I_125_** | 92.92 de | 96.97 b-d | 94.67 cd | 104.29 b | 113.45 a |  | **I_125_** | 26.92 b-f | 27.02 b-e | 27.20 c-e | 29.50 ab | 31.95 a |  | **I_125_** | 1.61 e-ı | 1.61 e-ı | 1.81 de | 2.02 bc | 2.27 a |
|  | | | | | | | | | | | | | | | | | | | |
| **Irrigation Level** | **Plant Height (cm)** | | | | |  | **Irrigation Level** | **Number of Leaves** | | | | |  | **Irrigation Level** | **Stalk Diameter (mm)** | | | | |
|  | **Fertilizer Doses** | | | | |  |  | **Fertilizer Doses** | | | | |  |  | **Fertilizer Doses** | | | | |
|  | **N_0_** | **N_80_** | **N_160_** | **N_240_** | **N_320_** |  |  | **N_0_** | **N_80_** | **N_160_** | **N_240_** | **N_320_** |  |  | **N_0_** | **N_80_** | **N_160_** | **N_240_** | **N_320_** |
| **I_0_** | 190.1 n | 191.3 n | 198.8 mn | 202.6 m | 212.8 l |  | **I_0_** | 6.41 k | 6.62 jk | 6.50 k | 6.98 ı-k | 7.23 hı |  | **I_0_** | 12.59 k | 12.68 jk | 12.73 jk | 13.06 ı-k | 13.09 h-k |
| **I_25_** | 219.7 l | 215.2 l | 233.8 k | 248.5 j | 247.2 j |  | **I_25_** | 7.13 ıj | 7.41 g-ı | 7.88 e-g | 8.05 ef | 8.40 de |  | **I_25_** | 12.55 k | 13.39 g-j | 13.27 g-k | 13.46 f-j | 14.30 b-e |
| **I_50_** | 241.8 jk | 257.6 ı | 267.3 h | 258.2 ı | 270.6 h |  | **I_50_** | 7.34 g-ı | 7.87 e-g | 8.17 ef | 8.36 d-f | 9.00 bc |  | **I_50_** | 13.62 e-h | 13.65 e-ı | 13.89 c-h | 13.92 c-g | 14.26 b-f |
| **I_75_** | 288.5 g | 272.9 h | 289.8 g | 293.6 e-g | 292.2 fg |  | **I_75_** | 7.78 f-h | 8.89 cd | 9.44 a-c | 9.45 a-c | 9.15 bc |  | **I_75_** | 13.77 d-ı | 13.93 c-g | 14.63 a-c | 14.35 b-e | 14.62 a-c |
| **I_100_** | 302.2 c-e | 297.0 d-g | 302.9 c-e | 307.3 bc | 300.6 c-f |  | **I_100_** | 8.83 cd | 9.07 bc | 9.36 a-c | 9.59 ab | 9.84 a |  | **I_100_** | 13.90 c-h | 14.54 a-d | 14.89 ab | 14.89 ab | 14.99 ab |
| **I_125_** | 302.3 c-e | 314.4 ab | 306.4 c-e | 305.5 b-d | 316.7 a |  | **I_125_** | 9.03 bc | 9.19 bc | 9.62 ab | 9.88 a | 9.88 a |  | **I_125_** | 14.03 c-g | 14.50 a-d | 15.00 ab | 15.18 a | 15.04 ab |
|  | | | | | | | | | | | | | | | | | | | |
| **Irrigation Level** | **Leaf/ Stalk Ratio (%)** | | | | |  | **Irrigation Level** | **Crude Protein (%)** | | | | |  | **Irrigation Level** | **Crude Ash (%)** | | | | |
|  | **Fertilizer Doses** | | | | |  |  | **Fertilizer Doses** | | | | |  |  | **Fertilizer Doses** | | | | |
|  | **N_0_** | **N_80_** | **N_160_** | **N_240_** | **N_320_** |  |  | **N_0_** | **N_80_** | **N_160_** | **N_240_** | **N_320_** |  |  | **N_0_** | **N_80_** | **N_160_** | **N_240_** | **N_320_** |
| **I_0_** | 10.41 l | 12.43 j-l | 13.14 h-k | 13.35 g-k | 12.80 ı-k |  | **I_0_** | 6.45 ef | 6.79 cd | 7.03 a-c | 7.23 a | 7.09 ab |  | **I_0_** | 5.84 j | 6.42 h-j | 6.45 h-j | 6.51 h-j | 6.11 ıj |
| **I_25_** | 11.21 kl | 14.52 e-j | 14.60 e-j | 15.97 c-f | 15.65 c-g |  | **I_25_** | 6.34 fg | 6.70 de | 7.07 ab | 7.16 a | 7.14 a |  | **I_25_** | 6.77 f-j | 7.44 c-h | 6.56 g-j | 7.32 d-h | 7.13 e-ı |
| **I_50_** | 12.50 ı-l | 15.38 d-h | 15.98 c-e | 16.71 a-e | 16.44 b-e |  | **I_50_** | 6.18 gh | 6.68 de | 7.14 a | 7.25 a | 7.16 a |  | **I_50_** | 7.49 c-h | 7.46 c-h | 7.67 b-g | 7.79 a-f | 7.55 c-h |
| **I_75_** | 13.68 f-j | 16.49 a-e | 17.53 a-d | 18.08 a-c | 17.35 a-d |  | **I_75_** | 6.16 gh | 6.50 ef | 7.09 ab | 7.21 a | 7.22 a |  | **I_75_** | 7.71 b-f | 8.21 a-e | 8.11 a-e | 7.82 a-f | 7.96 a-e |
| **I_100_** | 14.84 e-ı | 16.85 a-e | 17.70 a-d | 18.65 ab | 18.43 ab |  | **I_100_** | 5.94 h | 6.31 fg | 6.96 a-c | 7.23 a | 7.20 a |  | **I_100_** | 8.94 a | 8.03 a-e | 8.44 a-d | 8.57 a-c | 8.25 a-e |
| **I_125_** | 15.92 c-f | 16.70 a-e | 17.60 a-d | 17.62 a-d | 18.92 a |  | **I_125_** | 5.95 h | 5.96 h | 6.66 de | 6.82 b-d | 7.10 ab |  | **I_125_** | 8.84 ab | 8.55 a-c | 8.61 a-c | 8.48 a-d | 8.46 a-d |
|  | | | | | | | | | | | | | | | | | | | |
| **Irrigation Level** | **NDF (%)** | | | | |  | **Irrigation Level** | **ADF (%)** | | | | |  | **Irrigation Level** | **Phosphorus (%)** | | | | |
|  | **Fertilizer Doses** | | | | |  |  | **Fertilizer Doses** | | | | |  |  | **Fertilizer Doses** | | | | |
|  | **N_0_** | **N_80_** | **N_160_** | **N_240_** | **N_320_** |  |  | **N_0_** | **N_80_** | **N_160_** | **N_240_** | **N_320_** |  |  | **N_0_** | **N_80_** | **N_160_** | **N_240_** | **N_320_** |
| **I_0_** | 49.98 kl | 49.83 k-m | 48.50 lm | 47.88 mn | 46.37 n |  | **I_0_** | 28.93 j-l | 29.24 ı-l | 27.24 lm | 26.30 m | 25.83 m |  | **I_0_** | 0.060 m | 0.061 m | 0.075 kl | 0.099 c-g | 0.087 h-j |
| **I_25_** | 51.90 ıj | 53.60 e-j | 51.96 h-j | 48.01 l-n | 49.40 lm |  | **I_25_** | 29.63 ı-k | 30.09 h-j | 28.78 j-l | 27.25 lm | 27.60 k-m |  | **I_25_** | 0.079 j-l | 0.072 l | 0.074 kl | 0.100 b-g | 0.095 d-h |
| **I_50_** | 54.10 d-h | 52.21 g-j | 53.76 e-ı | 51.60 jk | 53.15 f-j |  | **I_50_** | 31.27 f-ı | 30.60 g-j | 31.09 f-ı | 29.96 h-j | 29.13 ı-l |  | **I_50_** | 0.075 kl | 0.100 b-g | 0.109 bc | 0.094 d-h | 0.097 d-h |
| **I_75_** | 54.78 d-f | 54.94 d-f | 55.32 c-f | 54.21 d-g | 52.31 g-j |  | **I_75_** | 32.29 d-g | 31.89 e-h | 32.97 b-f | 31.05 f-ı | 31.17 f-ı |  | **I_75_** | 0.099 c-g | 0.102 b-f | 0.101 b-g | 0.091 g-ı | 0.095 d-h |
| **I_100_** | 57.77 a | 53.65 e-j | 55.97 a-d | 57.13 a-c | 54.29 d-g |  | **I_100_** | 35.93 a | 33.22 b-f | 33.56 b-e | 34.40 a-d | 32.22 b-f |  | **I_100_** | 0.103 b-e | 0.125 a | 0.118 a | 0.103 b-d | 0.093 e-g |
| **I_125_** | 57.38 ab | 55.49 b-e | 55.21 c-f | 54.09 d-h | 54.79 d-f |  | **I_125_** | 34.83 ab | 34.57 a-c | 32.88 b-f | 32.83 b-f | 32.58 c-g |  | **I_125_** | 0.082 ı-k | 0.109 b | 0.094 d-h | 0.092 f-h | 0.095 d-h |
|  | | | | | | | | | | | | | | | | | | | |
| **Irrigation Level** | **Potassium (%)** | | | | |  | **Irrigation Level** | **Calcium (%)** | | | | |  | **Irrigation Level** | **Magnesium (%)** | | | | |
|  | **Fertilizer Doses** | | | | |  |  | **Fertilizer Doses** | | | | |  |  | **Fertilizer Doses** | | | | |
|  | **N_0_** | **N_80_** | **N_160_** | **N_240_** | **N_320_** |  |  | **N_0_** | **N_80_** | **N_160_** | **N_240_** | **N_320_** |  |  | **N_0_** | **N_80_** | **N_160_** | **N_240_** | **N_320_** |
| **I_0_** | 1.44 m | 1.53 l | 1.55 l | 1.59 kl | 1.67 ı-k |  | **I_0_** | 0.553 gh | 0.582 e-g | 0.475 j-l | 0.429 mn | 0.421 n |  | **I_0_** | 0.200 ı-l | 0.243 de | 0.173 n-p | 0.197 j-l | 0.208 h-k |
| **I_25_** | 1.53 l | 1.68 ı-k | 1.69 ı-k | 1.66 ı-k | 1.79 e-h |  | **I_25_** | 0.613 c-e | 0.788 a | 0.590 d-f | 0.473 j-l | 0.457 k-n |  | **I_25_** | 0.251 cd | 0.301 b | 0.138 q | 0.206 h-k | 0.197 j-l |
| **I_50_** | 1.61 j-l | 1.72 hı | 1.82 d-g | 1.75 f-ı | 1.79 e-h |  | **I_50_** | 0.609 c-f | 0.717 b | 0.633 c | 0.458 k-n | 0.449 k-n |  | **I_50_** | 0.250 cd | 0.301 b | 0.261 l-o | 0.213 g-ı | 0.175 m-p |
| **I_75_** | 1.74 g-h | 1.83 d-g | 1.92 b-d | 1.84 d-f | 1.86 c-e |  | **I_75_** | 0.607 c-f | 0.625 cd | 0.518 ıh | 0.481 jk | 0.476 j-l |  | **I_75_** | 0.175 m-p | 0.323 a | 0.184 l-o | 0.188 l-n | 0.190 k-m |
| **I_100_** | 1.87 c-e | 1.91 b-d | 1.97 ab | 2.02 a | 1.98 ab |  | **I_100_** | 0.593 d-f | 0.529 hı | 0.532 hı | 0.498 ıj | 0.463 j-m |  | **I_100_** | 0.164 p | 0.230 ef | 0.295 b | 0.170 op | 0.191 j-m |
| **I_125_** | 1.80 e-h | 1.84 d-f | 1.70 h-j | 1.95 a-c | 1.91 b-d |  | **I_125_** | 0.574 fg | 0.520 ıh | 0.478 jk | 0.445 k-n | 0.438 l-n |  | **I_125_** | 0.219 f-h | 0.224 fg | 0.196 j-l | 0.186 l-o | 0.186 l-o |

Supplemental Table 3. Effects of nitrogen doses and irrigation practices on leaf greenness, and stomatal conductance in sorghum. (The values in table are presented as the average of 2-year research results)

| **Irrigation Level** | **Leaf greenness (SPAD)** | | | | |  | **Irrigation Level** | **Stomatal Conductance (mmol m^-2^ s^-1^)** | | | | |
| --- | --- | --- | --- | --- | --- | --- | --- | --- | --- | --- | --- | --- |
|  | **Fertilizer Doses** | | | | |  |  | **Fertilizer Doses** | | | | |
|  | **N_0_** | **N_80_** | **N_160_** | **N_240_** | **N_320_** |  |  | **N_0_** | **N_80_** | **N_160_** | **N_240_** | **N_320_** |
| **I_0_** | 38.35 n | 43.01 l | 42.81 l | 42.60 l | 44.27 k |  | **I_0_** | 220.9 q | 227.9 pq | 239.3 o-q | 271.1 l-q | 238.6 o-q |
| **I_25_** | 40.17 m | 45.53 j | 48.04 hı | 45.72 j | 48.64 f-h |  | **I_25_** | 243.8 o-q | 253.4 n-q | 270.4 m-q | 299.6 ı-n | 273.8 l-p |
| **I_50_** | 47.54 ı | 47.74 hı | 48.38 g-ı | 50.92 c | 50.83 c |  | **I_50_** | 278.5 k-p | 288.9 j-o | 306.3 h-m | 339.5 f-j | 302.6 ı-n |
| **I_75_** | 49.16 e-g | 49.47 d-f | 49.55 d-f | 52.09 b | 52.41 ab |  | **I_75_** | 310.9 g-m | 303.4 ı-n | 325.9 f-k | 350.3 d-ı | 372.7 c-f |
| **I_100_** | 50.27 cd | 50.98 c | 51.09 c | 53.29 a | 53.14 ab |  | **I_100_** | 322.7 f-k | 354.9 d-h | 391.0 b-e | 406.1 bc | 372.1 c-f |
| **I_125_** | 49.79 de | 50.42 cd | 52.81 ab | 53.17 a | 52.87 ab |  | **I_125_** | 357.5 c-g | 402.7 b-d | 428.0 ab | 467.7 a | 431.7 ab |
